# Supplementary material for: A Critical View of the Application of the APEX Software (Aqueous Photochemistry of Environmentally-Occurring Xenobiotics) to Predict Photoreaction Kinetics in Surface Freshwaters
Source: Molecules. 2019 Dec 18;25(1):9. doi: 10.3390/molecules25010009 (PMC7017383; doi:10.3390/molecules25010009)
Supplement: Supplementary file 1 [file molecules-25-00009-s001.zip › 3.2.4_gcc-4.4.0.zip!/3.2.4_gcc-4.4.0/3.2.4_gcc-4.4.0/doc/octave/html/interpreter/Other-Sources.html]

Other Sources - Untitled


Previous: C++ Sources,
Up: Contributing Guidelines


---

### D.5 Other Sources

Apart from C++ and Octave language (m-files), Octave's sources include files
written in C, Fortran, M4, perl, unix shell, AWK, texinfo and TeX. There are
not many rules to follow when using these other languages; some of them are
summarized below. In any case, the golden rule is: if you modify a source
file, try to follow any conventions you can detect in the file or other similar
files.

For C you should obviously follow all C++ rules that can apply.

If you happen to modify a Fortran file, you should stay within Fortran 77
with common extensions like `END DO`. Currently, we want all sources
to be compilable with the f2c and g77 compilers, without special flags if
possible. This usually means that non-legacy compilers also accept the sources.

The M4 macro language is mainly used for autoconf configuration files. You should
follow normal M4 rules when contributing to these files. Some M4 files come
from external source, namely the Autoconf archive
http://autoconf-archive.cryp.to.

If you give a code example in the documentation written in texinfo with the
`@example` environment, you should be aware that the text within such an
environment will not be wrapped. It is recommended that you keep the lines
short enough to fit on pages in the generated pdf or ps documents. Here is a
ruler (in an `@example` environment) for finding the appropriate line
width:

```
              1         2         3         4         5         6
     123456789012345678901234567890123456789012345678901234567890
```
